# Maladie de Takayasu : une entité sous-diagnostiquée en Afrique Subsaharienne. À propos de cinq observations gabonaises

**DOI:** 10.48327/mtsi.v2i3.2022.272

**Published:** 2022-09-22

**Authors:** Josaphat IBA BA, Elsa AYO BIVIGOU, Christian ALLOGNON, Christelle AKAGHA, Obame Ulrich BEYEME BEYEME, Luis Felipe SOTO LORES, Jean Bruno BOGUIKOUMA

**Affiliations:** 1 Service de médecine interne, CHU de Libreville, Libreville, Gabon; 2 Service de cardiologie, CHU de Libreville, Libreville, Gabon; 3 Service de cardiologie, CHU d'Owendo, Libreville, Gabon; 4 Service de radiologie, CHU de Libreville, Libreville, Gabon

**Keywords:** Maladie de Takayasu, *Mycobacterium tuberculosis*, Corticoïdes, Méthotrexate, Gabon, Afrique subsaharienne, Takayasu's disease, *Mycobacterium tuberculosis*, Corticosteroid, Methotrexate, Gabon, Sub-Saharan Africa

## Abstract

La maladie de Takayasu est une vascularite affectant de façon préférentielle l'aorte et ses principales branches. *Mycobacterium tuberculosis* peut notamment être un facteur déclenchant du développement de la maladie de Takayasu par une réaction d'hypersensibilité. Le Maghreb et l'Afrique du Sud cumulent le plus grand nombre d'observations africaines, alors qu'entre les deux, en Afrique subsaharienne, les données parcellaires de la maladie font penser que la prévalence de la maladie est probablement sous-estimée/sous-diagnostiquée. Nous en rapportons 5 observations gabonaises.

## Abstract (en)

**Background:**

Takayasu's disease (TM) is a vasculitis of the aorta and its main branches, Infectious diseases, especially *Mycobacterium tuberculosis*, can be a trigger for the development of TM by a hypersensitivity reaction, but paradoxically very few cases of the disease are reported in the literature review.

**Materials and methods:**

Through a retrospective study carried out in the departments of internal medicine and cardiology of the Centre hospitalier universitaire de Libreville (CHUL) in Gabon, we identified all the observations of TM diagnosed in the said departments from 03/2014 to 05/2022, and rediscussed them on the basis of the criteria of Sharma *et al.* of 1996.

**Results:**

Five patients (3 men, 2 women), with an average age of 38.4 years were found. 2/5 patients reported a history of pulmonary tuberculosis. There was a change in general condition (n=4), fever (n=2), arterial hypertension (n=4), a decrease in the radial pulse (n=2), rheumatological manifestations (n=3), involvement of the serous membranes (n=3). CRP was positive (n=4), and HIV1,2 serology negative (n=5). Echocardiography found pulmonary arterial hypertension (n=1), constrictive pericarditis (n=1) and valvular involvement (minimal aortic insufficiency) (n=1), and typing of aortic involvement by computed tomography or CT angiography staged Takayasu's disease as type IIa (n=2), IIb (n=2) and IV (n=1). The patients were treated with oral corticosteroid therapy (n=5) combined with methotrexate (n=4) and azathioprine (n=1).

**Discussion:**

The criteria of Sharma *et al.* from 1996, suggest a high probability of TM with a sensitivity of 92.5% and a specificity of 95%. In sub-Saharan Africa, TM requires the elimination of certain differential diagnoses, particularly syphillis, tuberculosis, salmonellosis, and atherosclerosis. Corticosteroid therapy (coupled with immunosuppressive treatment) remains effective, allowing significant remission of the disease, but comes up against the risk of opportunistic infections.

**Conclusion:**

There is a contrast between the involvement of *Mycobacterium tuberculosis* in the etiopathogenesis of TM on the one hand and the endemic nature of tuberculosis on the African continent on the other hand; which contrasts with the few cases reported in sub-Saharan Africa.

## Introduction

La maladie de Takayasu (MT) encore dénommée « artérite aortique », « syndrome de l'arc aortique » et « maladie des femmes sans pouls », est une vascularite affectant les vaisseaux de gros calibre, particulièrement l'aorte et ses branches principales (artères sous-clavières, carotides, vertébrales, rénales, digestives et iliaques), mais également les artères coronaires et pulmonaires, et pouvant se compliquer d'anévrisme(s) et/ou de sténose(s) artérielle(s). Si les observations de cette maladie sont plus fréquentes au Japon, en Asie et en Amérique du Sud, la maladie de Takayasu existe en Afrique, mais est rarement rapportée dans sa littérature médicale, particulièrement subsaharienne [3, 13, 14, 15]. Nous rapportons 5 observations gabonaises de maladie de Takayasu diagnostiquées conjointement par les services de médecine interne et de cardiologie du CHU de Libreville.

## Matériel et Méthodes

Il s'agit d'une étude rétrospective, descriptive et analytique réalisée conjointement dans les services de médecine interne et de cardiologie du Centre hospitalier universitaire de Libreville (CHUL), prenant pour support les dossiers de patients ayant une maladie de Takayasu documentée, retenue sur la base des critères de Sharma *et al.* de 1996 (Tableau I) de 03/2014 à 05/2022. Les items de l’étude précisaient: les antécédents des patients, l'année de diagnostic, les signes pertinents cliniques et biologiques (vitesse de sédimentation, C-réactive protéine (CRP), hémogramme, créatinine, sérologie syphilitique (TPHAVDRL) et sérologie VIH1 et 2), les données morphologiques (tomodensitométrie/angioscanner thoracique et abdominal(e), échocardiographie), les données thérapeutiques en précisant les molécules utilisées et leur efficacité sur la symptomatologie clinique, et le devenir des patients.

**Tableau I:** Critères diagnostiques [20] Diagnostic criteria [20]

**Trois critères majeurs** Sténose ou occlusion de la portion moyenne de l'artère sous-clavière gauche en artériographie Sténose ou occlusion de la portion moyenne de l'artère sous-clavière droite en artériographie Symptômes caractéristiques d'une durée d'au moins 1 mois: claudication, abolition d'un pouls ou asymétrie tensionnelle, fièvre, cervicalgie, amaurose, troubles visuels, syncope, dyspnée, palpitations **Dix critères mineurs** Sensibilité des artères carotides à la palpation Pression artérielle brachiale > 140/90 mmHg ou pression artérielle poplitée > 160/90 mmHg Insuffisance aortique ou dilatation de l'anneau aortique Lésion des artères pulmonaires Sténose ou occlusion de la portion moyenne de la carotide gauche en artériographie Sténose ou occlusion du tiers distal du tronc brachio-céphalique en artériographie Lésion de l'aorte thoracique descendante en artériographie Lésion de l'aorte abdominale Lésion coronarienne avant 30 ans et en l'absence de dyslipidémie ou de diabète Vitesse de sédimentation >20 mm/heure

La présence de 2 critères majeurs, ou d'un critère majeur et 2 critères mineurs, ou de 4 critères mineurs, suggère une forte probabilité de maladie de Takayasu (sensibilité 92,5%, spécificité 95%)

## Observations

### Antécédents

Il s'agissait de 5 patients (sexe masculin n = 3, féminin n = 2, âge médian 38,4 ans (extrêmes 16 et 66)) avec 2 patients âgés de plus de 60 ans, et 3 patients de moins de 30 ans. Sur les 5 patients, 2 avaient déjà contracté une tuberculose pulmonaire 4 et 39 ans avant le diagnostic de MT, traitée pendant 6 mois – sous le protocole HRZE soit H: isoniazide (5 mg/kg/jour) + R: rifampicine (10 mg/kg/jour) + Z: pyrazinamide (30 mg/kg/jour) + E: éthambutol (20 mg/kg/jour)) pendant 2 mois suivi de 4 mois de HR (isoniazide (5 mg/kg/jour) + rifampicine (10 mg/kg/jour)) – et déclarés guéris.

### Caractéristiques générales

Les signes généraux étaient majoritairement marqués par une fièvre chez 2/5 patients, avec un état général majoritairement conservé (n = 4/5). Une hypertension artérielle existait dans 4/5 cas, précédant (n = 2) ou concomitant (n = 2) de sa survenue.

### Caractéristiques cliniques

Le pouls radial était diminué dans 2/5 cas, des manifestations rhumatologiques présentes dans 3/5 cas, de même qu'une atteinte d'au moins 2 séreuses dans 3/5 cas (Tableau II).

**Tableau II:** Antécédents et données cliniques des patients Patient history and clinical data

	Patient 1	Patient 2	Patient 3	Patient 4	Patient 5
**Profession**	Fonctionnaire retraité	Fonctionnaire retraité	Élève	Élève	Étudiante
**Année de diagnostic**	Mars 2022	D**é**cembre 2021	Mai 2019	Mars 2014	F**é**vrier 2019
**Âge (ans)**	64	66	19	16	27
**Antecedents**					
tuberculose	non	oui à 27 ans	oui à 14 ans	non	non
tabagisme	non	non	non	non	non
**Signes généraux**					
hypertension	oui (avant MT)	non	oui	oui (avant MT)	oui (concomitant)
fièvre	oui	oui	non	non	non
**é**tat g**é**n**é**ral	bon	alt**é**r**é**	bon	bon	bon
**Données cliniques**					
**Signes rhumatologiques**					
**Polyarthralgie**	oui	oui	non	non	non
petites articulations	oui	non			
grosses articulations	oui	oui			
**Atteinte des séreuses**					
plèvre	oui (bilat**é**rale)	oui (unilateral)	oui (bilaterale)	non	non
p**é**ricarde	oui	non	oui	non	non
p**é**ritoine	non	oui	oui	non	non
**Signes cutanés**	non	Nodules avant-bras et macules	non	non	
**Signes cardio-vasculaires**					
pouls	diminution au MS gauche	diminution pouls brachial G	diminution pouls radial G	diminution pouls radial G	normal

MT: maladie de Takayasu MS: membre supérieur G: gauche

### Caractéristiques biologiques et paracliniques (Tableaux III et IV)

**Tableau III:** Données biologiques des patients Patient biological data

Données biologiques	Patient 1	Patient 2	Patient 3	Patient 4	Patient 5
**CRP (mg/l)**	41,9	53,3	27,6	5	11
**VS (mm)**	50	130	99	22	30
**Hémogramme**					
leucocytes (/mm^3^)	12 000	29 090	5110	7520	6900
h**é**moglobine (g/dl)	8,4	9,4	11,3	12,8	13,2
plaquettes (/mm^3^)	259 000	340 000	453 000	312 000	231 000
**Creatinine (µmol/1)**	346	99	83	97	80
**VIH1,2**	n**é**gatif	n**é**gatif	n**é**gatif	négatif	négatif
**TPHA-VDRL**	négatif	négatif	négatif	négatif	non realise

CRP: C-réactive protéine VS: vitesse de sédimentation TPHA-VDRL: Treponema Pallidum Hemagglutinations Assay - Veneral Disease Research Laboratory

**Tableau IV:** Données morphologiques des patients Patient morphological data

	Patient 1	Patient 2	Patient 3	Patient 4	Patient 5
**TDM/angioscanner thoracique et abdominal**					
calcifications athéromateuses	oui (pariétales de l'artére sous-clavière gauche, crosse de l'aorte, aorte thoracique descendante)	oui (aorte et ses branches, carotide commune, carotide interne, et sous-clavière bilatérale)	irrégularités pariétales évoquant des micro-anévrismes aortiques, calcifications péricardiques	oui (aorte thoracique descendante et à l'origine ASC gauche)	sténose aorte abdominale avec extension au niveau du tronc cœliaque et en infrarénal
sténose	non	non	non	origine sous-clavière G, et de 58% (aorte descendante) (Fig. 1)	oui
anévrisme	non	non	oui	oui (jonction aorte thoracique et aorte abdominale)	
classification topographique	type IIb	type IIa	type IIa	type IIb	type IV
**Échocardiographie**					
fraction dejection	80%	70%	59%	75%	72%
pression artère pulmonaire (mmHg)	normale	normale	78	normale	normale
valvulopathie	absence	insuffisance aortique	absence	absence	absence
autres pathologies associées	non	leucémie myéloïde chronique	péricardite constrictive et cœur pulmonaire chronique	non	non
**Critères diagnostiques de Sharma *et al* 1996**	2 critères majeurs 3 critères mineurs	2 critères majeurs 2 critères mineurs	1 critère majeur 2 critères mineurs	2 critères majeurs 3 critères mineurs	4 critères mineurs

TDM: tomodensitométrie ASC: artère sous clavière

**Figure 1:**
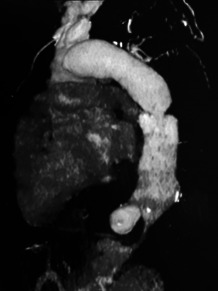
Image de reconstruction de sténose de l'aorte descendante (Patient N° 4) Descending aortic stenosis reconstruction image (Patient no 4)

Un syndrome inflammatoire (CRP et vitesse de sédimentation) existait dans 4/5 cas. À l'hémogramme prédominait une normocytose dans 3 cas, une anémie constante (extrêmes: 8,4 à 11,3 g/dl) et des plaquettes toujours normales. Il existait une atteinte rénale avec clearance de la créatininémie à 346 μmol/l (n = 1), une protéinurie/24 heures néphrotique (n = 1) à 8,4 grammes et néphrétique (n = 1) à 1 gramme. Les sérologies VIH1 et 2 étaient négatives. Sur le plan paraclinique le diagnostic de MT était confirmé à l'angioscanner par l'existence de calcifications athéromateuses de l'aorte chez tous nos patients, avec 2 sténoses et 2 lésions d'anévrisme à chaque fois pour des patients distincts. Il existait une calcification d'une (n = 2) ou des 2 artères sous-clavières (n = 1). À l’échocardiographie existait dans 1 cas une hypertension artérielle pulmonaire (n = 1), une péricardite constrictive (n = 1) et une atteinte valvulaire (insuffisance aortique minime) (n = 1). Le typage des atteintes de l'aorte par tomodensitométrie ou angioscanner stadifiait la maladie de Takayasu en type IIa (n = 2), IIb (n = 2) et IV (n = 1).

### Évolution sous traitement et données de suivi (Tableau V)

**Tableau V:** Données thérapeutiques et évolutives des patients Patient therapeutic and evolutionary data

**Traitement**					
prednisone (relais)	1 mg/kg/jour	1 mg/kg/jour	1 mg/kg/jour	1 mg/kg/jour	0,7 mg/kg/jour
methotrexate (mg/semaine)	non	15	non	non	20
azathioprine (mg/jour)	150 mg/jour	non	non	non	
**Devenir**					
favorable	oui	oui pendant 2 mois			
Décès		a 4 mois du diagnostic par tuberculose pulmonaire compliquée de dissection carotide commune	a 12 mois du diagnostic d'insuffisance cardiaque terminale	
Perdus de vue					oui
Poursuite de suivi	oui			oui	

Tous les patients avaient bénéficié d'une corticothérapie orale à la posologie de 1 mg/kg/jour, associée à un traitement immunosuppresseur: méthotrexate (n = 4) à la posologie de 15 mg/semaine, ou azathioprine (n = 1) à la posologie de 150 mg/jour. Deux patients décédaient à 4 mois et à 12 mois du diagnostic, respectivement après une évolution favorable se compliquant à 2 mois de l'initiation d'un traitement immunosuppresseur (corticothérapie + méthotrexate) de tuberculose pulmonaire, compliquée de dissection de la carotide commune pour le premier, et d'une insuffisance cardiaque droite terminale pour le second. Deux patients étaient régulièrement revus, et une patiente perdue de vue.

## Discussion

La première observation de MT a été rapportée par Mikito Takayasu en 1908 sous la dénomination d'artérite de Takayasu, et ce n'est qu'en 1954 que la terminologie actuelle de maladie de Takayasu a été proposée. Les présentations cliniques de la maladie sont variables et sous dépendance de poussées inflammatoires systémiques et de retentissement de complications de la maladie (sténose et anévrisme particulièrement). Toutefois, des cas asymptomatiques ont été documentés, généralement découverts de manière fortuite [12]. Les données pathogéniques actuelles de la MT sont mieux connues à travers l'exploration in situ des lésions artérielles résultant des différents prélèvements chirurgicaux vasculaires. Les lésions vasculaires de Takayasu contiennent des macrophages, des cellules lymphoïdes (lymphocytes T αß CD4+ et CD8+, les cellules γδ, cellules naturelles tueuses, et lymphocytes B). Les infiltrats inflammatoires à proximité des vasa vasoranéoangiogéniques seraient probablement la porte d'accès à la paroi artérielle. La cible de la réponse immunitaire reste insaisissable, mais les données suggèrent la présence locale d'antigènes vasculitogènes, avec amorçage et maintien de la réponse immunitaire dans la paroi artérielle [2].

Sharma *et al.* [20], qui retiennent 2 critères majeurs, ou 1 critère majeur et 2 critères mineurs, ou 4 critères mineurs, suggèrent une forte probabilité de maladie de Takayasu avec une sensibilité de 92,5% et une spécificité de 95%. Nos patients ont un nombre de critères diagnostiques suffisants pour le diagnostic de MT [20].

Au Gabon à ce jour, une seule observation de MT chez un adulte jeune a été rapportée dans la littérature [10]. Deux de nos patients signalaient l'existence de tuberculose antérieure traitée et guérie 4 et 39 ans avant le diagnostic de MT. Les maladies infectieuses, en particulier à *Mycobacterium tuberculosis*, peuvent être un déclencheur du développement de la MT par une réaction d'hypersensibilité. En revanche, l'inflammation de l'aorte en résultant peut être secondaire à l'invasion directe de l'artère par *Mycobacterium tuberculosis* [11].

Nos patients cumulaient certaines données cliniques de la phase systémique ou préocclusive marquée par des manifestations générales (fièvre, arthralgies, altération de l’état général, signes pleuro-pulmonaires), et de la phase occlusive caractérisée par un retentissement en périphérie des sténoses. Ces premiers symptômes n’étant pas spécifiques, ils rendent le diagnostic précoce de la MT difficile.

Nous retrouvions de façon prédominante une atteinte des séreuses avec au moins 2/3 séreuses chez 4 de nos patients. Deux péricardites avaient été retrouvées: une isolée, l'autre associée à une atteinte pleurale et péritonéale avec association à des calcifications péricardiques. Cette atteinte des séreuses peut parfois se retrouver comme mode de révélation de la MT. Hamzaoui *et al.* [7] dans la revue de la littérature retrouvaient des observations de péricardite exsudative au cours de la MT, d’évolution favorable sous corticothérapie, la rattachant de ce fait à l'expression de la maladie. La pleurésie était dans notre série retrouvée dans 3/5 cas. Elle est rapportée dans la littérature par différents auteurs dont Gui *et al.* (dont une méta-analyse retrouvait également 3 pleurésies unilatérales) [6], et Achari *et al.* [1]. L'ascite rarement décrite dans la revue de la littérature existait dans 2/5 cas, retrouvée chez un patient présentant une péricardite constrictive, et chez un autre une leucémie myéloïde chronique (LMC) stable. Cette association de la LMC et de la MT, peu retrouvée, est la seconde de la littérature après celle de Yuce Inel *et al.* [23]. L'hypertension artérielle retrouvée chez 4/5 de nos patients, fait partie des signes de la phase vasculaire où prédomine la symptomatologie ischémique créée par les lésions artérielles responsable de l'expression clinique variée de la maladie: hypertension artérielle, claudication des membres inférieurs ou supérieurs, ischémie cérébrale, angor. L'hypertension artérielle dans la MT a pour étiologie: une insuffisance artérielle rénale, une pseudo-coarctation aortique et rigidité pariétale secondaire à l'insuffisance vasculaire, une augmentation de la pression du pouls lors d'insuffisance valvulaire aortique [18].

Dans nos explorations cardiologiques, une atteinte valvulaire a été dépistée comme rapportée par Ren *et al.* [17], de même qu'une hypertension artérielle pulmonaire comme Yang *et al.* [22].

Les manifestations rhumatologiques se résumaient à une atteinte polyarticulaire prédominant sur les grosses articulations, intéressant nos 2 patients les plus âgés d'un âge supérieur à 60 ans.

La biologie ne comporte aucun test spécifique de la MT, son seul intérêt est d'authentifier un syndrome inflammatoire. De même l'histologie n'est pas nécessaire pour le diagnostic, elle est habituellement réalisée sur des pièces opératoires lorsqu'une chirurgie de correction est retenue. En accord avec la classification topographique de 1994 (conférence de consensus de Tokyo 1994, 16), nous avons classé nos observations en type IIa (n = 2), IIb (n = 2) et IV (n = 1). Dans la revue de la littérature, la mesure de la distance intima-média permet d'apprécier l'importance de la plaque d'athérome, dont l’âge élevé et le sexe masculin sont les facteurs déterminants de majoration de l’épaisseur intima-média [9]. Malheureusement, cette mesure n'a pas été systématiquement réalisée.

Le diagnostic différentiel principal de la MT est l'artérite à cellules géantes (ACG) touchant également les vaisseaux de gros calibre. L'ACG affecterait plus fréquemment les sujets d'origine nord-européenne, tandis que l'observation clinique selon laquelle la MT pourrait être plus fréquente dans les populations d'ascendance asiatique ou africaine doit être confirmée par des études épidémiologiques. Des associations distinctes d'antigènes leucocytaires humains de classe II ont été identifiées comme facteurs de risque génétiques d'ACG et de MT [5]. Les autres diagnostics différentiels de la MT sont toutes les autres causes d'aortite et d'atteintes de la paroi aortique: aortites infectieuses (syphilis, tuberculose, salmonellose) et inflammatoires (lupus érythémateux systémique, polyarthrite rhumatoïde, maladie de Behçet), l'athérosclérose et les dysplasies fibromusculaires [16]. Sur le plan thérapeutique, la corticothérapie est recommandée dans les formes actives de la maladie à la dose de 0,5 mg/kg/jour dans les formes moyennes limitées à la paroi artérielle, et à la dose de 0,7 à 1 mg/kg/jour (sans dépasser 70 mg/jour) dans les formes plus sévères de la maladie (retentissement sur les organes vitaux, atteintes artérielles multiples et/ou évolutives, hypertension rénovasculaire, insuffisance rénale vasculaire, atteinte des coronaires, ischémie d'un membre, infarctus cérébral secondaire à une artérite des troncs supra-aortiques, ischémie digestive symptomatique, insuffisance aortique) [18]. Ce recours à la corticothérapie est d'autant plus utile et efficace que le diagnostic est porté tôt en phase pré-occlusive, permettant une rémission de la maladie dans 25 à 50% des cas [14].

Concernant l'adjonction à un immunosuppresseur dans un but d’épargne cortisonique, nous avons majoritairement pris comme alternative le méthotrexate (4/5 de nos patients) comme recommandé dans la littérature [18].

Dans les pays où les biothérapies sont d'actualité, infliximab, etanercept, tocilizumab, abatacept ont été proposes dans la MT avec de très bons résultats [21]. Toutefois, nos résultats peuvent être jugés satisfaisants sans le recours à ces agents ni au mycophénolate mofétil. Ce qui souligne l'intérêt qu'un diagnostic précoce offre l'opportunité d'un traitement plus rapide, éventuellement moins prolongé du fait de lésions moins inflammatoires.

**Figure 2:**
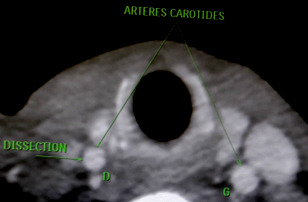
Dissection de la carotide commune (Patient N° 2) Common carotid dissection (Patient no 2)

**Figure 3:**
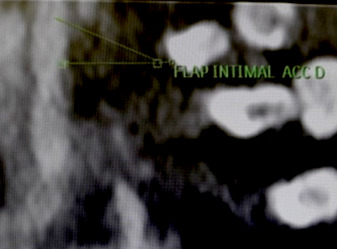
Dissection de la carotide commune (Flap intimal carotide commune droite) (Patient N° 2) Common carotid dissection flap intima (Patient no 2)

Concernant le devenir des patients, nous déplorons deux décès: un chez un patient présentant des problèmes de compliance au traitement et chez qui la MT s'associait à une péricardite constrictive, et l'autre une MT associée à une leucémie myéloïde chronique documentée dont l’évolution s'est vue compliquée de réactivation de tuberculose pulmonaire et de dissection de la carotide commune (Fig. 2, 3). Cette dissection de la carotide commune a été rapportée au cours de la MT par certains auteurs [8], mais reste encore mal comprise sur le plan de sa physiopathologie. Certaines études ont suggéré que les patients atteints de dissection artérielle pourraient avoir une faiblesse génétiquement déterminée de la paroi des vaisseaux, et que des facteurs environnementaux, tels qu'une infection aiguë (comme dans notre cas) ou un traumatisme mineur, pourraient servir de déclencheurs [4, 19].

## Conclusion

Les données concernant la MT en Afrique subsaharienne demeurent parcellaires. Tenant compte de l'implication du *Mycobacterium tuberculosis* dans l’étiopathogénie de cette maladie d'une part, et du caractère endémique de la tuberculose sur le continent africain d'autre part, qui contraste avec le peu de cas rapportés, nous pouvons dire que la prévalence de cette affection en Afrique subsaharienne est probablement sous-estimée.

## Liens D'intérêts

Les auteurs ne déclarent aucun lien d'intérêt.

## Contribution des Auteurs

Josaphat IBA BA: conception, rédaction. Elsa AYO BIVIGOU: rédaction, relecture. Christian ALLOGNON: recherche bibliographique, relecture. Christelle AKAGHA: relecture, collecte de données. Luis Felipe SOTO LORES: collecte des données radiologiques, relecture. Obame Ulrich BEYEME BEYEME: recherche bibliographique, relecture. Jean Bruno BOGUIKOUMA: conception, relecture, approbation de la version finale.
